# Supplementary material for: Prostanoid Metabolites as Biomarkers in Human Disease
Source: Metabolites. 2022 Aug 4;12(8):721. doi: 10.3390/metabo12080721 (PMC9414732; doi:10.3390/metabo12080721)
Supplement: Supplementary file 1 [file metabolites-12-00721-s001.zip › metabolites-1841386-supplementary.pdf]

Supplementary Table S1. Molecular identity and frequently used synonym names in the literature.

PubChem ID and Structure	Metabolite	Synonyms used in the literature
5280360<br>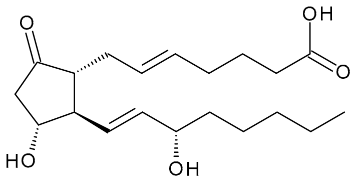	**PGE_2**<br><br>MF: $C_{20}H_{32}O_5$<br>MW: 352.5 g/mol	• IUPAC Name:<br>(Z)-7-[(1R,2R,3R)-3-hydroxy-2-[(E,3S)-3-hydroxyoct-1-enyl]-5-oxocyclopentyl]hept-5-enoic acid
5280711<br>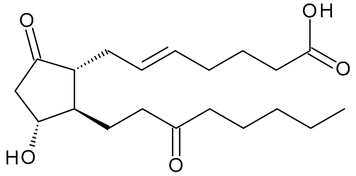	**13,14-dihydro-15-keto PGE_2**<br><br>MF: $C_{20}H_{32}O_5$<br>MW: 352.5 g/mol	• IUPAC Name:<br>(Z)-7-[(1R,2R,3R)-3-hydroxy-5-oxo-2-(3-oxooctyl)cyclopentyl]hept-5-enoic acid<br><br>• 9,15-dioxo-11α-hydroxy-prost-5Z-en-1-oic acid<br>• 15-keto-13,14-dihydro-PGE_2<br>• 15-keto-13,14-dihydroprostaglandin E_2<br>• PGEM (NB: both 13,14-dihydro-15-keto PGE_2 and Tetranor-PGEM) are referred to as PGEM)
161468<br>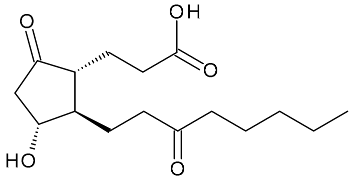	**Tetranor-PGEM**<br><br>MF: $C_{16}H_{24}O_7$<br>MW: 328.36 g/mol	• IUPAC Name:<br>8-[(1r,2r,5r)-2-(2-Carboxyethyl)-5-hydroxy-3-oxocyclopentyl]-6-oxooctanoic acid<br><br>• 9,15-dioxo-11α-hydroxy-13,14-dihydro-2,3,4,5-tetranor-prostan-1,20-dioic acid<br>• 7α-hydroxy-5,11-diketotetranor-prosta-1,16-dioic acid<br>• PGE-MuM<br>• PGEM (NB: both 13,14-dihydro-15-keto PGE_2 and Tetranor-PGEM) are referred to as PGEM)

5283043<br>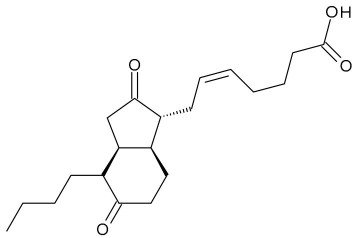	**Bicyclo PGE_2**<br><br>MF: $C_{20}H_{30}O_4$<br>MW: 334.4 g/mol	• IUPAC Name:<br>(Z)-7-[(1R,3aS,7aS)-4-butyl-2,5-dioxo-3,3a,4,6,7,7a-hexahydro-1H-inden-1-yl]hept-5-enoic acid<br><br>• 11-deoxy-13,14-dihydro-15-keto-11β,16χ-cycloprostaglandin E_2
5283042<br>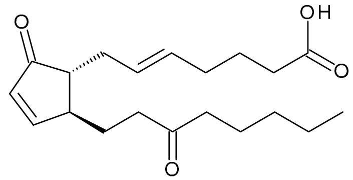	**13,14-dihydro-15-keto PGA_2**<br><br>MF: $C_{20}H_{30}O_4$<br>MW: 334.4 g/mol	• IUPAC Name:<br>(Z)-7-[(1R,5S)-2-oxo-5-(3-oxooctyl)cyclopent-3-en-1-yl]hept-5-enoic acid<br><br>• 9,15-dioxo-prosta-5Z,10-dien-1-oic acid<br>• 15-keto-13,14-dihydro-PGA_2
448457<br>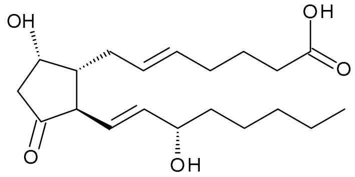	**PGD_2**<br><br>MF: $C_{20}H_{32}O_5$<br>MW: 352.5 g/mol	• IUPAC Name:<br>(Z)-7-[(1R,2R,5S)-5-hydroxy-2-[(E,3S)-3-hydroxyoct-1-enyl]-3-oxocyclopentyl]hept-5-enoic acid
44600448<br>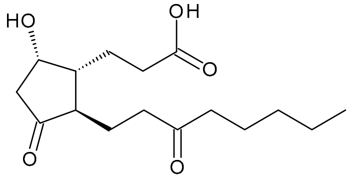	**Tetranor-PGDM**<br><br>MF: $C_{16}H_{24}O_7$<br>MW: 328.36 g/mol	• IUPAC Name:<br>8-[(1R,2R,3S)-2-(2-carboxyethyl)-3-hydroxy-5-oxocyclopentyl]-6-oxooctanoic acid<br><br>• 9S-hydroxy-11,15-dioxo-2,3,4,5-tetranor-prostan-1,20-dioic acid

5283036<br>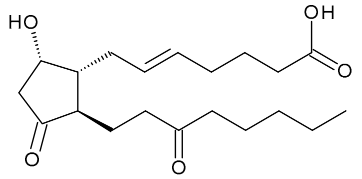	**13,14-dihydro-15-keto PGD$_2$**<br><br>MF: $C_{20}H_{32}O_5$<br>MW: 352.5 g/mol	• IUPAC Name:<br>(Z)-7-[(1R,2R,5S)-5-hydroxy-3-oxo-2-(3-oxooctyl)cyclopentyl]hept-5-enoic acid<br><br>• 9*α*-hydroxy-11,15-dioxo-prost-5Z-en-1-oic acid<br>• dhk-PGD$_2$
5280886<br>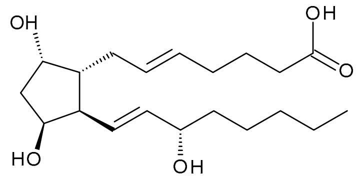	**11β-PGF$_{2\alpha}$**<br><br>MF: $C_{20}H_{34}O_5$<br>MW: 354.5 g/mol	• IUPAC Name:<br>(Z)-7-[(1R,2R,3S,5S)-3,5-dihydroxy-2-[(E,3S)-3-hydroxyoct-1-enyl]cyclopentyl]hept-5-enoic acid<br><br>• (5Z,9*α*,11β,13E,15S)-9,11,15-trihydroxy-prosta-5,13-dien-1-oic acid<br>• 11-epi-PGF$_{2\alpha}$<br>• 9α,11β-PGF$_2$
5280884<br>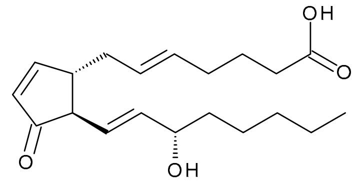	**PGJ$_2$**<br><br>MF: $C_{20}H_{30}O_4$<br>MW: 334.4 g/mol	• IUPAC Name:<br>(Z)-7-[(1S,5R)-5-[(E,3S)-3-hydroxyoct-1-enyl]-4-oxocyclopent-2-en-1-yl]hept-5-enoic acid<br><br>• 11-oxo-15S-hydroxy-prosta-5Z,9,13E-trien-1-oic acid<br>• 9-deoxy-Δ^9-prostaglandin D$_2$
5280885<br>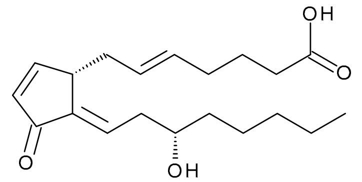	**Δ12-PGJ$_2$**<br><br>MF: $C_{20}H_{30}O_4$<br>MW: 334.4 g/mol	• IUPAC Name:<br>(*Z*)-7-[(1*S*,5*E*)-5-[(3*S*)-3-hydroxyoctylidene]-4-oxocyclopent-2-en-1-yl]hept-5-enoic acid<br><br>• 11-oxo-15S-hydroxy-prosta-5Z,9,12E-trien-1-oic acid

5283052<br>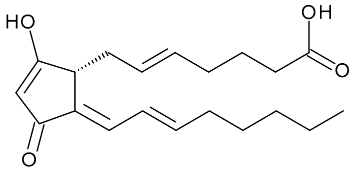	**15-deoxy-Δ^{12,14}-PGD$_2$**<br><br>MF: $C_{20}H_{30}O_4$<br>MW: 334.4 g/mol	• IUPAC Name:<br>(Z)-7-[(1R,2E,5S)-5-hydroxy-2-[(E)-oct-2-enylidene]-3-oxocyclopentyl]hept-5-enoic acid<br><br>• 9α-hydroxy-11-oxo-prosta-5Z,12E,14E-trien-1-oic acid<br>• 15-deoxy-PGD$_2$<br>• 15dPGD$_2$
5311211<br>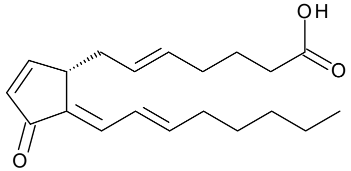	**15-deoxy-Δ^{12,14}-PGJ$_2$**<br><br>MF: $C_{20}H_{28}O_3$<br>MW: 316.4 g/mol	• IUPAC Name:<br>(Z)-7-[(1S,5E)-5-[(E)-oct-2-enylidene]-4-oxocyclopent-2-en-1-yl]hept-5-enoic acid<br><br>• 11-oxo-prosta-5Z,9,12E,14E-tetraen-1-oic acid<br>• 15-deoxy-PGJ$_2$<br>• 15dPGJ$_2$
5280363<br>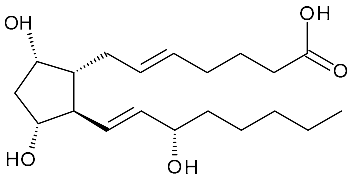	**PGF$_{2\alpha}$**<br><br>MF: $C_{20}H_{34}O_5$<br>MW: 354.5 g/mol	• IUPAC Name:<br>(Z)-7-[(1R,2R,3R,5S)-3,5-dihydroxy-2-[(E,3S)-3-hydroxyoct-1-enyl]cyclopentyl]hept-5-enoic acid
5283039<br>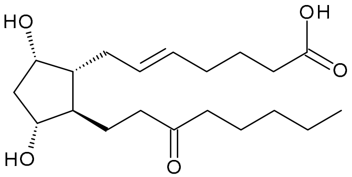	**13,14-dihydro-15-keto PGF$_{2\alpha}$**<br><br>MF: $C_{20}H_{34}O_5$<br>MW: 354.5 g/mol	• IUPAC Name:<br>(Z)-7-[(1R,2R,3R,5S)-3,5-dihydroxy-2-(3-oxooctyl)cyclopentyl]hept-5-enoic acid<br><br>• 9α-hydroxy-11,15-dioxo-prost-5Z-en-1-oic acid<br>• Dhk-PGF$_{2\alpha}$<br>• 15-keto-13,14-dihydro-PGF$_{2\alpha}$<br>• PGFM (NB: both 13,14-dihydro-15-keto PGF$_{2\alpha}$ and Tetranor-PGFM are referred to as PGFM)

3082337<br>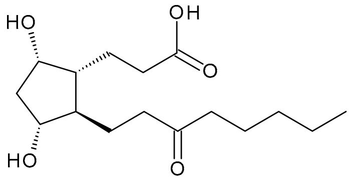	**Tetranor-PGFM**<br><br>MF: $C_{16}H_{26}O_7$<br>MW: 330.37 g/mol	• IUPAC Name:<br>8-[2-(2-carboxyethyl)-3,5-dihydroxycyclopentyl]-6-oxooctanoic acid<br><br>• 9,11-Dppcpa<br>• U-PGFM<br>• PGFM (NB: both 13,14-dihydro-15-keto $PGF_{2\alpha}$ and Tetranor-PGFM are referred to as PGFM)
5280497<br>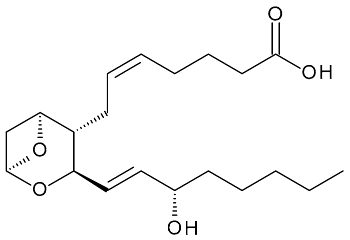	**TXA_2**<br><br>MF: $C_{20}H_{32}O_5$<br>MW: 352.5 g/mol	• IUPAC Name:<br>(Z)-7-[(1S,3R,4S,5S)-3-[(E,3S)-3-hydroxyoct-1-enyl]-2,6-dioxabicyclo[3.1.1]heptan-4-yl]hept-5-enoic acid
5283137<br>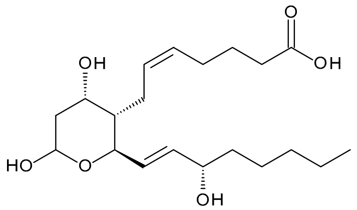	**TXB_2**<br><br>MF: $C_{20}H_{34}O_6$<br>MW: 370.5 g/mol	• IUPAC Name:<br>(Z)-7-[(2R,3S,4S)-4,6-dihydroxy-2-[(E,3S)-3-hydroxyoct-1-enyl]oxan-3-yl]hept-5-enoic acid<br><br>• 9α,11,15S-trihydroxythromba-5Z,13E-dien-1-oic acid
5280891<br>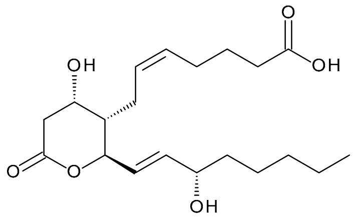	**11-dehydro-TXB_2**<br><br>MF: $C_{20}H_{32}O_6$<br>MW: 368.5 g/mol	• IUPAC Name:<br>(Z)-7-[(2R,3S,4S)-4-hydroxy-2-[(E,3S)-3-hydroxyoct-1-enyl]-6-oxooxan-3-yl]hept-5-enoic acid<br><br>• 9α,15S-dihydroxy-11-oxothromba-5Z,13E-dien-1-oic acid<br>• 11-keto-thromboxane B_2<br>• 11-dh-TXB_2<br>• TXBM

5283138<br>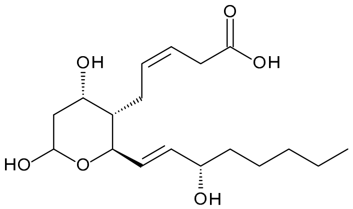	**2,3-dinor-TXB$_2$**<br><br>MF: $C_{18}H_{30}O_6$<br>MW: 342.4 g/mol	• IUPAC Name:<br>(Z)-5-[(2R,3S,4S)-4,6-dihydroxy-2-[(E,3S)-3-hydroxyoct-1-enyl]oxan-3-yl]pent-3-enoic acid<br><br>• 2,3-dinor-thromboxane B$_2$<br>• 9S,11,15S-trihydroxy-2,3-dinor-thromboxa-5Z,13E-dien-1-oic acid
5282411<br>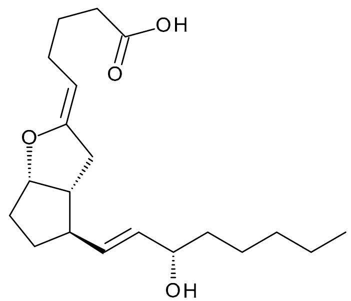	**PGI$_2$**<br><br>MF: $C_{20}H_{32}O_5$<br>MW: 352.5 g/mol	• IUPAC Name:<br>(5Z)-5-[(3aR,4R,5R,6aS)-5-hydroxy-4-[(E,3S)-3-hydroxyoct-1-enyl]-3,3a,4,5,6,6a-hexahydrocyclopenta[b]furan-2-ylidene]pentanoic acid
5280888<br>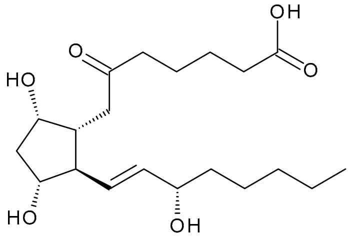	**6-keto-PGF$_{1\alpha}$**<br><br>MF: $C_{20}H_{34}O_6$<br>MW: 370.5 g/mol	• IUPAC Name:<br>7-[(1R,2R,3R,5S)-3,5-dihydroxy-2-[(E,3S)-3-hydroxyoct-1-enyl]cyclopentyl]-6-oxoheptanoic acid<br><br>• 6-oxo-9α,11α,15S-trihydroxy-prost-13E-en-1-oic acid<br>• 6-k-PGF$_{1\alpha}$<br>• 6-oxo-PGF$_{1\alpha}$<br>• PGIM (NB: both 6-keto-PGF$_{1\alpha}$ and 2,3-dinor-6-keto-PGF$_{1\alpha}$ are referred to as PGIM)

5283084 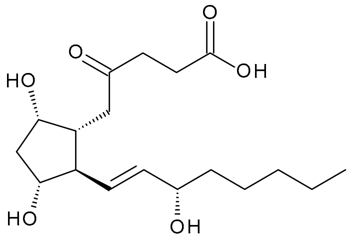	**2,3-dinor-6-keto-PGF$_{1\alpha}$**<br><br>MF: $C_{18}H_{30}O_6$<br>MW: 342.4 g/mol	• IUPAC Name:<br>• 5-[(1R,2R,3R,5S)-3,5-dihydroxy-2-[(E,3S)-3-hydroxyoct-1-enyl]cyclopentyl]-4-oxopentanoic acid<br><br>• 6-oxo-9α,11α,15S-trihydroxy-2,3-dinor-prost-13E-en-1-oic acid<br>• 2,3-Dk- PGF$_{1\alpha}$<br>• 2,3-dinor-6-oxo-PGF$_{1\alpha}$<br>• PGI$_2$-M<br>• u-PGIM<br>• PGIM (NB: both 6-keto-PGF$_{1\alpha}$ and 2,3-dinor-6-keto-PGF$_{1\alpha}$ are referred to as PGIM)
